# Development, Status Quo, and Challenges to China’s Health Informatization During COVID-19: Evaluation and Recommendations

**DOI:** 10.2196/27345

**Published:** 2021-06-17

**Authors:** Mian Huang, Jian Wang, Stephen Nicholas, Elizabeth Maitland, Ziyue Guo

**Affiliations:** 1 Dong Fureng Institute of Economic and Social Development Wuhan University Beijing China; 2 Center for Health Economics and Management School of Economics and Management Wuhan University Wuhan China; 3 Australian National Institute of Management and Commerce Sydney Australia; 4 Newcastle Business School University of Newcastle Newcastle Australia; 5 Guangdong Institute for International Strategies Guangdong University of Foreign Studies Guangzhou China; 6 School of Economics Tianjin Normal University Tianjin China; 7 School of Management Tianjin Normal University Tianjin China; 8 School of Management University of Liverpool Liverpool United Kingdom

**Keywords:** health informatization, COVID-19, health policy, digital health, health information technology, China

## Abstract

By applying advanced health information technology to the health care field, health informatization helps optimize health resource allocation, improve health care services, and realize universal health coverage. COVID-19 has tested the status quo of China’s health informatization, revealing challenges to the health care system. This viewpoint evaluates the development, status quo, and practice of China’s health informatization, especially during COVID-19, and makes recommendations to address the health informatization challenges. We collected, assessed, and evaluated data on the development of China’s health informatization from five perspectives—health information infrastructure, information technology (IT) applications, financial and intellectual investment, health resource allocation, and standard system—and discussed the status quo of the internet plus health care service pattern during COVID-19. The main data sources included China’s policy documents and national plans on health informatization, commercial and public welfare sources and websites, public reports, institutional reports, and academic papers. In particular, we extracted data from the 2019 National Health Informatization Survey released by the National Health Commission in China. We found that China developed its health information infrastructure and IT applications, made significant financial and intellectual informatization investments, and improved health resource allocations. Tested during COVID-19, China’s current health informatization system, especially the internet plus health care system, has played a crucial role in monitoring and controlling the pandemic and allocating medical resources. However, an uneven distribution of health resources and insufficient financial and intellectual investment continue to challenge China’s health informatization. China’s rapid development of health informatization played a crucial role during COVID-19, providing a reference point for global pandemic prevention and control. To further promote health informatization, China’s health informatization needs to strengthen top-level design, increase investment and training, upgrade the health infrastructure and IT applications, and improve internet plus health care services.

## Introduction

### Background

**Figure 1:**
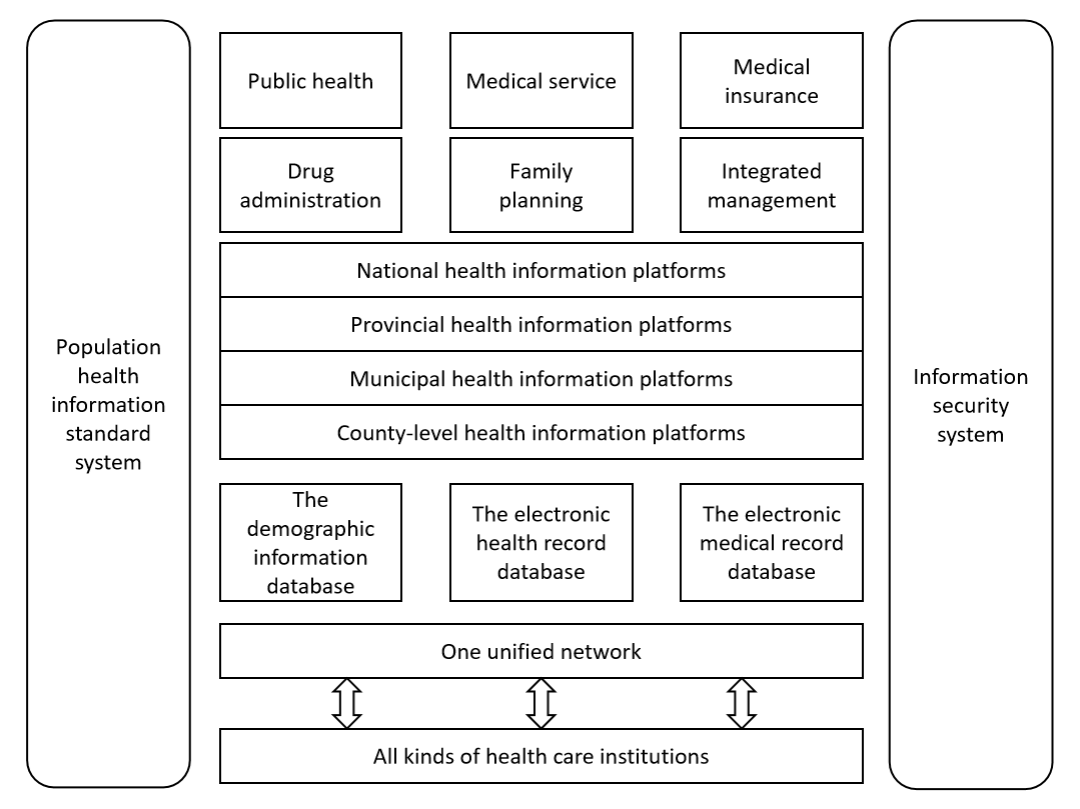
The “4631-2 framework” of health informatization.

Building on China’s highly developed information-based society, including big data, cloud computing, mobile internet, and artificial intelligence, health information technology (HIT) provides a key impetus for the Chinese government to maximize health resource allocation, address the uneven geographical distribution of medical resources, ensure China’s universal health coverage, and enable health care providers to optimize health care services with lower medical costs and better quality [1-6]. China’s health informatization has developed through several stages, and the policy system at every stage has sought consistency and coordination. In 2010, the National Health and Family Planning Commission (NHFPC) issued the “3521 framework” for health informatization, which was then upgraded to the “4631-2 framework” in 2013 [7-10]. Figure 1 presents a structural figure of the “4631-2 framework” based on NHFPC policy documents. As shown in Figure 1, the “4631-2 framework” referred to 4 levels of national health information platforms (national, provincial, municipal, and county level), 6 primary applications (public health, medical service, medical insurance, drug administration, family planning, and integrated management), 3 major health information databases (the demographic information database [DID], the electronic health record database [EHRD], and the electronic medical record database [EMRD]), 1 unified network covering all kinds of health care institutions (including traditional Chinese medical institutions), and 2 systems (population health information standard system and information security system) [8,11].

Building on the “4631-2 framework” of health informatization, the 2016 Plan of Healthy China 2030 committed to expanding the health information service system. The NHFPC released the National Population Health Informatization Development Plan in 2017, emphasizing the essential role of health informatization in national IT (information technology) construction, health reform and the Healthy China initiative [12]. In 2018, the National Health Commission (NHC) was established, further emphasizing the role of health informatization by targeting comprehensive and lifelong intelligent health services by utilizing advanced technologies.

### Objective

Surprisingly, the development of China’s health informatization system has not been detailed nor its status quo effectiveness assessed. The early COVID-19 period, where, for example, the extensive use of internet plus health care, including internet hospitals and telemedicine, helped to ensure the normal operation of the medical system, provides a unique opportunity to evaluate China’s health informatization [13-15]. Our study aimed to map the development of China’s health informatization; evaluate China’s status quo health informatization experiences and lessons learned during COVID-19; identify the challenges to health informatization; and provide recommendations for the improvement of health informatization.

## Data Sources and Approach

The NHC conducted a 2018 survey on national health informatization, which was published in the 2019 National Health Informatization Survey Report [16]. The NHC also held several expert meetings to assess the survey data, which was also published in the survey report [16]. The data in the NHC Survey Report can be divided into two parts: regional health informatization and hospital informatization. The Regional Health Informatization Survey covered 1650 health commissions (HCs), including all 32 provincial HCs, 69.6% (296/425) of municipal HCs, and 47.1% (1322/2803) of county-level HCs. The Hospital Informatization Survey covered 5470 hospitals, including 70.1% (1480/2112) of tertiary hospitals, 62.1% (3730/6004) of tertiary hospitals, and 6.2% (260/4180) of other hospitals (at lower levels). As shown in Multimedia Appendix 1, the survey identified key aspects of health informatization, including infrastructure, IT application, capital investment, and human resources, providing data for evaluating the status quo of China’s health informatization. Multimedia Appendix 1 also displays other information we collected, interpreted, and evaluated from primary sources, academic papers, government reports, and institutional reports regarding the current status and challenges of health informatization.

Figure 1 sets out a diagrammatic representation of the conceptual basis of China’s health informatization, while Multimedia Appendix 1 summarizes the main primary and secondary sources used to outline the development, evaluate the status quo, and identify the challenges to China’s health informatization.

## Development and Status Quo of China’s Health Informatization

### Health Information Infrastructure

China’s health information platforms form the basis of the country’s regional health informatization. In 2012, the Ministry of Health in China proposed a 3-level (national, provincial, and regional) health information platform [9]. As displayed in Figure 1, the health information platform was extended in 2013 to consist of 4 administrative level platforms: the national, provincial, municipal, and county-level information platforms [10]. On each platform, the medical data from different regions were integrated and shared [7]. The platforms were designed to be unified, authoritative, and interconnected so as to exploit and statistically analyze health information of the whole population, thus providing support for management and decision making on a national health level [12,17,18]. The platforms also provided the basis for an information business system covering the whole industry chain of health and medical big data, linking medical care, medical insurance, and medicine. By 2018, 93.8% (30/32) of provincial health information platforms, 66.2% (196/296) of municipal health information platforms, and 48.2% (637/1322) of county-level health information platforms had been established [16]. During COVID-19, by sharing data with internet hospitals and health care institutions at all levels, regional health information platforms connected online and offline services to provide patients with full process services before, during, and after diagnosis [19,20]. During the COVID-19 period, the NHC promoted new infrastructure construction in health informatization, emphasizing the need to improve health information platforms and health information databases [19].

National health information databases include three major databases: the DID, the EHRD, and the EMRD. The DID contains fundamental population information, family planning service management information, and the nonresident population management information. The EMRD stores all information from electronic medical records while the EHRD holds residents’ personal health information from the electronic health record (EHR), which is defined as “digitally stored health care information about an individual’s lifetime with the purpose of supporting continuity of care, education, and research and ensuring confidentiality at all times” [7,21]. In recent years, the NHC has promoted the EHR to be opened to individuals. In pilots undertaken in selected areas, residents are able to enter the EHR system with a password and access their own EHRs [22,23]. The 2010 “3521 framework” contained only two databases—the EHRD and the EMRD [24]—with DID added under the 2013 “4631-2 framework” [25].

The three major databases are relatively independent but interrelated. Supported by the national health information platform, information in the three databases is shared and dynamically updated to ensure consistency, accuracy, and integrity of the information [12,26]. According to the 2019 National Health Informatization Survey, the average construction rate of EHRDs was 79.5% (1312 out of 1650 HCs had built EHRDs), the highest among the three databases, with 93.8% (30/32) of provincial, 84.8% (251/296) of municipal, and 78.0% (1031/1322) of county-level EHRDs. The average construction rate of the DIDs was 77.5% (1278 out of 1650 HCs), with 100% (32/32) of provincial, 80.4% (238/296) of municipal, and 76.2% (1008/1322) of county-level DIDs. Lowest among these three databases, the average construction rate of EMRD was 49.5% (816 out of 1650 HCs), which was 93.8% (30/32) of provincial, 59.5% (176/296) of municipal, and 46.1% (610/1322) of county-level EMRDs [16].

### IT Applications

**Figure 2:**
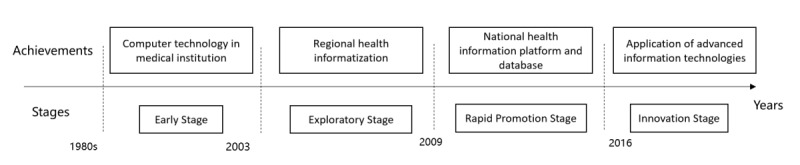
The development history of health informatization in China.

To illustrate the historical development of technology applications, we constructed Figure 2, which sets out the four development stages in China’s health informatization: the early stage from the 1980s to 2003, the exploratory stage from 2003 to 2009, the rapid development stage from 2009 to 2016, and the innovation stage since 2016 [27,28]. In the early stage (1980s-2003), the emphasis was placed on the application of computer technology in information systems, such as the financial management system and charging system, in large medical institutions. Manual operations were replaced by computer functions in the management of traditional businesses, including financial management, fee management, and drug management. At the exploratory stage (2003-2009), drawing on the experience of SARS (severe acute respiratory syndrome) in 2003, China strengthened its informatization in health emergency command, disease prevention and control, and public health resources and health information management [29]. Importantly, a direct internet reporting system for infectious diseases and public health emergencies was established, and regional health informatization emerged. In the context of health care reform, the rapid development stage (2009-2016) completed the construction of the national health information platform and database, dealing with the information islands in health services as well as realizing the platform connectivity and information sharing of the medical and health systems. Since 2016, more advanced information technologies, such as internet plus, big data, cloud computing and artificial intelligence have been widely applied to health industries. In the innovation stage, a people-centered medical service principle was adopted [30], with advanced IT applications improving the health information system and providing more diverse health information services [12].

Currently, advanced technologies commonly used in China’s health informatization include cloud computing, big data, Internet of Things, mobile internet, and artificial intelligence. The 2019 National Health Informatization Survey showed that advanced technologies, especially mobile internet and big data, were more commonly applied on provincial than municipal and county platforms. In 2018, on provincial health information platforms, the application rate of cloud computing was 68.8% (22 out of 32 provinces), big data was 53.1% (17 out of 32 provinces), Internet of Things was 21.9% (7 out of 32 provinces), mobile internet was 71.9% (23 out of 32 provinces), and artificial intelligence was 12.5% (4 out of 32 provinces). On municipal and county-level platforms, advanced technologies were less utilized, where 40.2% (119/296) of municipal and 46.1% (609/1322) of county-level platforms had not utilized any advanced technologies [16]. Higher-level hospitals had higher IT application rates, with mobile internet being the most commonly used technology (tertiary hospitals: 747/1480, 50.5%; secondary hospitals: 767/3730, 20.6%; and other hospitals: 63/260, 24.2%) and artificial intelligence being the least used technology (tertiary hospitals: 137/1480, 9.3%; secondary hospitals: 62/3730, 1.7%; and other hospitals: 3/260, 1.2%).

COVID-19 tested the informatization of China’s health system. In COVID-19 prevention and control, advanced information technologies were effectively utilized in monitoring and forecasting the pandemic’s trends as well as constraining the spread of the virus. Based on big data, the intelligent risk assessment system and automatic early warning system were utilized to fight the virus. Big data was used to track the mobility of the population and locate crowds after the outbreak. With the help of artificial intelligence, the number of infections was estimated, and high-risk areas identified, which helped the government to implement timely control methods and determine the allocation of resources [31]. Using a series of data statistics and model analysis, big data population movement information on Baidu Maps was one of the most popular technologies to estimate the number and locations of patients with COVID-19 and carriers. The big data methods also predicted disease trends based on real-time reporting of new cases.

In order to improve data utilization, some hospitals promoted the connection between hospital information systems and regional health information platforms. Hospital information departments extracted data from major information systems such as Hospital Information System, Laboratory Information System, Picture Archiving and Communication Systems, and EMRs to build a clinical data center [32,33]. An induction model based on artificial intelligence was constructed to help predict the risk and trend of infectious diseases and enhance prevention efforts [1].

### Financial and Intellectual Investment

As seen in Multimedia Appendix 1, the importance of health informatization can be assessed by China’s increased levels of investment. During the 2011-2015 12th Five-Year Plan, the central government invested more than ¥10 billion (US $1.5 billion) into health informatization [7]. In 2017, the first phase of the National Health Security Informatization Project was approved by the National Development and Reform Commission, with ¥340 million (US $52.5 million) invested in national platform construction and 32 national hospital informatization projects approved by the NHFPC, and ¥310 million (US $47.9 million) invested by the NHFPC in the construction of information platforms and clinical data centers [34]. In 2018, the Ministry of Finance invested ¥670 million (US $103.5 million) to equip 832 poor county hospitals and 1664 primary-level medical institutions with telemedicine equipment. In 2019, the central government allocated ¥180 million (US $27.8 million) to increase technology applications, including the internet, big data, and artificial intelligence in 9 provinces, with the aim of improving the diagnosis and treatment capacity of family doctors [35].

Although governmental investment in health informatization expanded rapidly, it failed to cover China’s current financial informatization needs, reflecting a lack of stable and sustainable capital input. For example, China’s health expenditure accounted for 7.56% of the financial expenditure, while health informatization expenditures accounted for only 0.7% of the total health expenditure [36]. Second, there were obvious regional differences in governmental investment, with Beijing, Guangzhou, Shanghai, and the eastern coastal provinces achieving rapid development of health informatization versus the less developed provinces [37]. The capital input in informatization was positively associated with the local economic development level, where informatization tended to increase regional health inequality [16]. The 2019 National Health Informatization Survey showed that hospital informatization investment was limited. The proportion of informatization investment in a hospital’s annual revenue was between 0.1% and 1% in 67.3% (3681/5470) of hospitals and 1% to 5% in 23.7% (1296/5470) of hospitals, with only 3.7% (202/5470) of hospitals investing over 5% of their annual revenue in informatization. With the construction of health informatization requiring multisectoral collaboration, the lack of social supervision led to a lack of initiative and a waste of resources. In 2017, according to the China Hospital Information Management Association survey, hospital self-raised funds accounted for 68.8% of the total investment in hospital informatization, with government financial funds accounting for 28.4% and other funds accounting for 2.8% of the total investment [38,39]. Although the Chinese government has implemented policies to encourage enterprise initiative, an incentive mechanism to encourage social capital to participate in health informatization construction has not been set up in China. Measures to promote government-enterprise cooperation in health informatization are still being explored [12,40].

Both the quantity and quality of technical informatization personnel failed to meet demand [12]. The number of health informatization personnel was small. In 2019, the average staff number in provincial information departments was only 20, while 91.6% (271/296) of municipal information departments and 93.6% (1238/1322) of county-level information departments had less than 9 informatization staff. In hospitals, the information department of tertiary hospitals had on average 10 staff, while 74.1% (2764/3730) of secondary hospitals and 89.5% (233/260) of other hospitals had less than 4 informatization staff. In particular, there was a lack of highly educated and specialized informatization personnel and an uneven distribution of high-quality personnel in administration and hospitals at different levels [41]. High-level administrations and hospitals tended to have staff with higher education [16], with the proportion of staff with a bachelor’s degree or above in provincial information departments (88.9%) and in municipal information departments (83.8%) significantly higher than that in county-level information departments (51.6%). In tertiary hospitals, 67.5% of information department staff had bachelor’s degrees, while in secondary and other hospitals, most staff had degrees below the junior college level. The proportion of staff with a master’s degree or above in tertiary hospitals (14.3%) was significantly higher than that in secondary hospitals (2.3%) and in other hospitals (0.7%). Staff specialized in computer science accounted for 85.1% of informatization staff in tertiary hospitals compared to 49.1% in secondary and 26.5% in other hospitals. In addition, IT staff with senior professional titles were scarce in all types of hospitals, with only 2.0% in tertiary, 1.9% in secondary, and 0.6% in other hospitals [16].

### Health Resource Allocation

Medical resources distribution and health service capacity were unevenly distributed regionally and by type of medical institution [3,4]. The data from Multimedia Appendix 1 reveals that the construction strategies of health informatization varied according to the health care resource allocation status in different regions [1].

For developed regions (mainly the eastern coast) with abundant medical resources, the local health administration undertook the major responsibilities for integrating high-quality medical resources and promoting health informatization. These regions had stronger abilities to independently develop informatization and provide stable and sufficient funds for informatization. Bottom-up building of health information platforms and data centers were established by the local health administration to connect the information system of health institutions at all levels in the administrative region, achieving the sharing and exchange of medical information. For example, municipal and county-level health commissions in each province independently carried out informatization construction, driving the development of provincial health informatization and promoting the development of regional health information infrastructures, information technologies, and professionals. Municipal and county-level health resource information was integrated, and the connectivity of health information within the province was realized [24].

In developing regions, mainly in western and central China, where medical resources were scarce, large hospitals generally connected with several local primary health care institutions to realize medical information exchange and sharing [24]. For instance, the West China Hospital of Sichuan University cooperated with surrounding secondary hospitals and community health service centers to achieve regional medical collaboration. Shengjing Hospital of China Medical University, in cooperation with public hospitals at all levels in Liaoning and Shenyang, constructed the Shengjing Medical Alliance, a nonprofit medical cooperative alliance. In these less developed and health resource scarce regions, unified management was implemented within the medical alliances to integrate medical resources at different levels. To optimize medical resources, resource-poor regions utilized telemedicine, or the use of telecommunications technologies to provide medical information and services, [1,4,42] for remote consultation and two-way referral, where primary medical units can get support from large hospitals and gain access to high-quality medical resources and services.

The COVID-19 pandemic typically led to a surge in demand for medical care, which overwhelmed local capabilities [43,44]. During the pandemic, telemedicine played an increasingly important role in medical resource allocation [1,4,42]. As the worst-hit area of COVID-19 in China, Hubei Province’s medical institutions were overloaded with a severe shortage of medical resources and services [43]. Due to the implementation of control measures such as lockdowns, traffic control, and community closure management, residents’ access to health care resources was severely restricted. Telemedicine was widely used to overcome geographical barriers and expand the supply of medical resources [45-47]. With the aid of informatization technologies, many doctors from other parts of China provided remote diagnosis and treatment services. For instance, the medical team from Guangdong Province built an internet hospital to provide online clinical support for patients in Jingzhou, Hubei Province. More than 1300 doctors from 15 medical institutions in Guangdong participated, with the total number of online visitors exceeding 100,000 within 18 days of its launch, making up for local medical service deficits in Hubei Province [48].

### The Standard System

As seen in Figure 1 and Multimedia Appendix 1, emphasis has been placed on the standardization of health information, or the standard system [12]. In 2006, the Special Committee on Health Information Standards was approved by the Ministry of Health, where work on health information norms were standardized. Started in 2012, the National Medical Health Information System’s Interconnection and Interoperability Standardization Certification [16] aimed to strengthen the management and promote the implementation of health information standards, so as to achieve health information interoperability [11]. The Regional Health Informatization Survey and Hospital Informatization Survey found that by 2017, 54 regional platforms (1 provincial platform, 45 municipal platforms, and 8 county-level platforms), and 90 hospitals (84 tertiary hospitals and 6 secondary hospitals) had been certified. Between 2018 and 2019, the number certified significantly increased with an additional 48 regional platforms and 101 hospitals certified [49].

The security system of regional health information platforms contained 4 levels: physical security, network security, system security, and application security. Physical security required hardware security and the geographical location security of the computer room and key infrastructure. Network security included link redundancy, firewall setup, and intrusion detection. System security included data backup, virus prevention, operating system security, and vulnerability checks. Application security included identity authentication and authority management [8,50]. By 2018, all provincial platforms, 90.9% (269/296) of municipal platforms, and 78.9% (1043/1322) of county-level platforms had formulated an information security system. Meanwhile, 99.2% (1468/1480) of tertiary hospitals, 95.9% (3577/3730) of secondary hospitals, and 81.5% (212/260) of other hospitals had formulated an information security system. By August 2020, with 227 effective information standards approved, the standard system for regional health informatization and hospital informatization had been basically established throughout the health system [26].

During COVID-19, continuing shortcomings in the standard system were addressed by the 2020 Notice on Strengthening the National Health Information Standardization System, which defined four key tasks for the construction of the national health information standardization system: (1) to promote the standardization of the national health information infrastructure, including the national health information platform, national hospital information platform, government services integration platform, and to standardize the information from grassroots health institutions, public health systems, and traditional Chinese medicine hospitals; (2) to strengthen the standardization of the DID, the EHRD, the EMRD, and the health care resource database; (3) to promote the standardization of advanced IT applications, comprising five technologies: internet plus health care, health and medical big data, health care artificial intelligence, health care 5G technology, and blockchain technology for health care; and (4) to improve the standardization of network security, namely industry network security standard system, data security standard, and industry application security standard. In order to improve the disease prevention and control system and enhance the ability to respond to public health emergencies, the NHC formulated the National Public Health Informatization Construction Standard and Specification on December 11, 2020, to further regulate national public health informatization, specifying 21 first-level indicators, 125 second-level indicators, and 421 third-level indicators of health service management and IT application content in all types of health institutions [26].

Even with these reforms, shortcomings in China’s health information standard system remain. There was a lack of standard evaluation, and the consistency and interoperability of the standard system needed strengthening [1]. The formulation of China’s standard system lagged behind the development of new technologies. The safety standards, data standards, functional standards, quality assessment standards for information technology products, and management standards for data quality were incomplete. In terms of information security standards, the ownership and usage rights of patients’ health information, as well as the division of rights and responsibilities among all parties, needed further clarification in laws and regulations. There was a lack of security standard evaluation. The Regional Health Informatization Survey reported that in 2018, 75.0% (24/32) of provincial platforms, 30.4% (90/296) of municipal platforms, and 17.7% (234/1322) of county-level platforms had implemented the evaluation of classified information security protection. Only 46.4% (687/1480) of tertiary hospitals, 18.6% (694/3730) of secondary hospitals, and 10% (26/260) of other hospitals had participated in the evaluation. Although China continues to develop health information standards, due to the large number of departments involved, many standards and norms were not unified. There were also problems related to unclear management responsibility for the standards and ineffective supervision mechanism [11].

### Status Quo of the Internet Plus Health Care Service Pattern During COVID-19

**Figure 3:**
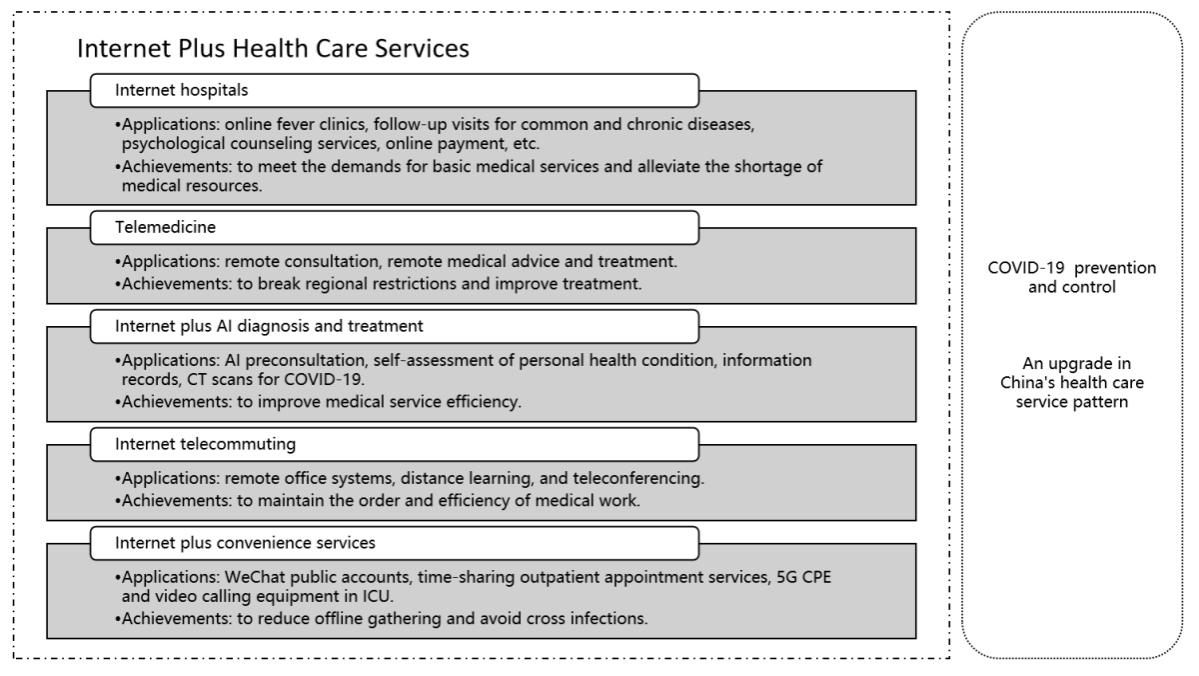
The applications and achievements of internet plus health care in COVID-19. AI: artificial intelligence, CT: computed tomography, CPE: customer premise equipment, ICU: intensive care unit.

As shown in Figure 3, the new service pattern of internet plus health care sought to meet increasing medical demands and virus prevention and control needs during the COVID-19 pandemic.

Internet plus health care has the characteristics of remoteness, efficiency, intelligence, and convenience, providing online consultation, remote diagnosis, psychological counseling, chronic disease follow-up, and drug distribution to meet people’s medical demands [40,48]. During COVID-19, internet plus health care aimed to break through time and space constraints to both identify and service infected people, block transmission routes, and reduce the risk of population infection. The popularization of internet plus health care has not only improved the efficiency of health care institutions at all levels [51] but also stimulated innovation and accelerated the upgrading of China’s health care service pattern. We set out below the contours of internet plus health care during the COVID-19 pandemic.

#### Internet Hospitals: To Meet the Demands for Basic Medical Services and Alleviate the Shortage of Medical Resources During COVID-19

Figure 3 identifies the role of internet hospitals and online fever clinics after the COVID-19 outbreak, which were widely established to provide follow-up visits for common and chronic diseases, as well as for psychological counseling services. In an internet hospital, a full set of medical services such as registration, consultation, diagnosis, and prescription were completed by means of video, voice, and graphic communication [52]. Hospitals arranged the delivery of drugs while patients made online payments [53]. By effectively serving a large number of patients with COVID-19, internet hospitals reduced diagnosis and treatment pressure in brick-and-mortar hospitals, greatly alleviating the shortage of medical resources in severely affected COVID-19 areas.

#### Telemedicine: To Break Regional Restrictions and Improve Treatment During COVID-19

As specified in Figure 3, remote consultation through the telemedicine platform integrated high-quality medical resources and provided support from large institutions and well-known experts to improve treatment. Due to the uneven distribution of medical resources in different regions, China attached great importance to constructing a telemedicine system, which played a particularly significant role during the pandemic. As discussed above, a typical case was the establishment of 9 telemedicine centers in Guangdong Province to support the hard-hit COVID-19 city of Jingzhou. While on-site diagnosis was carried out, remote medical advice and treatment were provided via the telemedicine platform at the same time.

#### Internet Plus Artificial Intelligence Diagnosis and Treatment: To Improve Medical Service Efficiency During COVID-19

As shown in Figure 3, online artificial intelligence preconsultation functions were widely used to assess COVID-19 risks and provide emotional care. People made a self-assessment of their personal health condition with the help of technologies powered by artificial intelligence, where patients provided their personal information, past medical history, and current medical conditions through dialogue before seeing a doctor. This information was recorded by artificial intelligence and integrated into the health information system, then directly added into the patient’s medical record to facilitate subsequent diagnosis. Artificial intelligence was also applied to CT (computed tomography) scans for COVID-19, improving the accuracy and efficiency of front-line medical work [31].

#### Internet Telecommuting: To Maintain the Order and Efficiency of Medical Work During COVID-19

As noted in Figure 3, distance learning and teleconferencing had become mainstream working modes during the pandemic. Many hospitals used virtual private network (VPN) technology to establish secure data links, and combined with virtual desktop infrastructure technology, a remote office environment was built. By opening the VPN account and private cloud desktop, the remote office system enabled medical staff to log into intranet workstations to work remotely [54].

To enhance the service ability of medical staff, distance education was also used widely during the pandemic. Learning content was issued on online learning platforms. To check the effectiveness of these learning platforms, medical staff were required to take regular online tests, and test results were recorded in real time. Moreover, IT companies such as Tencent and Alibaba launched free online meeting systems to support antipandemic work, enabling the pandemic prevention working groups to arrange work, and discuss diagnosis methods and pandemic prevention countermeasures, through video conferencing. It not only reduced crowd gathering but also improved hospital management efficiency.

#### Internet Plus Convenience Services: To Reduce Offline Gathering and Avoid Cross Infections During COVID-19

During the pandemic, many hospitals provided internet plus convenience services for the public, as specified in Figure 3. Through the publication function of WeChat public accounts, hospitals launched real-time news and science articles about COVID-19 as part of information campaigns, which prevented rumors and helped the public acquire accurate pandemic knowledge [14,43]. In addition, many hospital information departments opened time-sharing outpatient appointment services, where the hospital’s WeChat public account allowed patients to enter the appointment and registration system according to patients’ personal needs. The appointment service helped separate patients’ arrival time and reduced the number of people in the doctor’s waiting room at one time, containing the spread of the virus [53]. To facilitate communication between intensive care unit (ICU) patients and their families, while avoiding staff gathering, some hospitals installed 5G customer premise equipment and video calling equipment in ICUs, substituting 5G mobile video for physical visits during COVID-19.

China’s internet plus service pattern also played an important role for overseas Chinese people, providing authoritative and scientific knowledge of COVID-19 prevention. The platform was launched on April 7, 2020, bringing together 26 well-known Chinese medical institutions and third-party service institutions to provide free internet consulting services for overseas Chinese individuals. On April 30, a special column aimed at overseas students was launched to provide free health consultation and psychological counseling services. By September 2020, the platform had been visited more than 150 million times, with more than 6 million people providing direct consulting services [48].

## Challenges and Recommendations

### Challenges Ahead

According to the findings presented above, significant challenges to China’s health informatization remain. Table 1 classifies and summarizes the challenges to health informatization. First, the number of informatization professionals is small, and the distribution of human resources is unbalanced. Second, the investment from government and medical institutions is insufficient to support the development of health informatization. An incentive mechanism to encourage social capital to participate in health informatization construction has not been set up in China [3,28]. Third, the IT application on health information platforms and in hospitals at all levels remains low. Fourth, the information standards and security systems are incomplete.

**Table 1:** Challenges to national health informatization.

Factors			Descriptions
**Personnel**			
	Quantity	The number of senior technical personnel and grassroots practical personnel is insufficient	
	Structure	Human resource allocation as measured by quality, educational background, professional title, and age is unbalanced	
**Funds**			
	Financial	Financial support is generally insufficient and shows regional differences	
	Institutional	Low proportion of hospitals’ annual revenue allotted to informatization investment	
	Social	A lack of social capital’s participation and government-enterprise cooperation	
**IT^a^ applications**			
	Regional health information platform	Low application rate on municipal and county-level information platforms	
	Hospital information system	Low application rate in secondary hospitals and primary hospitals	
**Systems**			
	Information standard system	A lack of system interoperability and interconnection, standard management, and supervision	
	Information security system	A lack of complete and specific systems for standard evaluation and health information security protection	

^a^IT: information technology.

### Recommendations

Below we present recommendations to further promote health informatization in China.

#### Strengthen Top-Level Design

As identified in Table 1, there is a need to strengthen top-level design. Legal restrictions on some key aspects of national health informatization, such as health care big data management, internet medical information services, privacy protection, information facilities security, and network security, need to be enhanced. The information standard and security systems should be designed in more unified and specific ways, and information security supervision needs to be strengthened [55]. An incentive mechanism should be set up to encourage a diverse range of organizations and institutions, including health care institutions, research institutes, universities, associations, and enterprises, to participate in the construction of health informatization.

#### Increase Financial Investment

As specified in Table 1, finance, taxation, and investment arrangements need to be increased to support health informatization, especially from the central government and especially in regions with limited financial resources for health informatization. To address informatization financial constraints, public-private partnerships should be expanded to strengthen the cooperation between the government, business enterprises, and various social institutions [28].

#### Increase Qualified Personnel

As shown in Table 1, there is an urgent need to increase qualified personnel specializing in HIT. An integrated and comprehensive training system should be built to cover junior college, undergraduate, graduate, and continuing education students to enlarge the scale of skilled workers. An incentive mechanism should be established to attract experienced professionals and maintain existing personnel in health information departments. To improve the professional level and comprehensive quality of health information personnel, measures should include establishing a national health big data research institute jointly with universities and research institutes, setting up health information majors in colleges and universities and carrying out continuing education to combine teaching content with practical work.

#### Promote IT Applications

Addressing the deficits in information platforms and systems identified in Table 1 requires the reallocation of health resources and promotion of advanced technologies to support the new service pattern of internet plus health care. Advanced IT applications, such as cloud computing, big data, mobile internet, and artificial intelligence, need to be popularized in grassroots health units. It is necessary to construct more internet hospitals, building an integrated online and offline medical service model covering prediagnosis, in-treatment, and postdiagnosis to improve health care service efficiency.

## Conclusions

As shown by its crucial role in COVID-19 prevention and control, health informatization was “fit for purpose” in China. Developed over the past decade, health information platforms and health information databases facilitated data sharing between health care institutions at all levels, connecting online and offline services. Advanced information technologies, including cloud computing, big data, and artificial intelligence, effectively monitored and forecasted the pandemic’s trend that facilitated health authorities’ COVID-19 control and prevention ability. The new service pattern of internet plus health care broke through time and space constraints, reduced cross infections, improved medical service quality and efficiency [51], alleviated the shortage of medical resources, and met the increasing medical demands during COVID-19. However, China’s health informatization is a work in progress; informatization is incomplete and the uneven regional distribution of health resources continues to persist. To address the challenges to health informatization, we recommend the strengthening of top-level design, increasing investment and training, upgrading the health infrastructure and IT applications, and improving internet plus health care services.

## Appendix

Multimedia Appendix 1 Data sources on the development and status quo of China’s health informatization from government, commercial, and public welfare sources and websites; academic papers; public reports; institutional reports; and fieldwork.

## Abbreviations

CT: computed tomography
DID: demographic information database
EHR: electronic health record
EHRD: electronic health record database
EMRD: electronic medical record database
HC: health commission
HIT: health information technology
ICU: intensive care unit
IT: information technology
NHC: National Health Commission
NHFPC: National Health and Family Planning Commission
SARS: severe acute respiratory syndrome
VPN: virtual private network

## References

[1] Liang J, Zheng X, Chen Z, Dai S, Xu J, Ye H, Zhang Z, Ge F, Lei J (2019). The experience and challenges of healthcare-reform-driven medical consortia and Regional Health Information Technologies in China: A longitudinal study. Int J Med Inform.

[2] Li L, Fu H (2017). China's health care system reform: Progress and prospects. Int J Health Plann Manage.

[3] Yao H, Zhan C, Sha X (2020). Current situation and distribution equality of public health resource in China. Arch Public Health.

[4] (2009). Central Committee and the State Council, People's Republic of China. Opinions on Deepening the Reform of the Health Care System.

[5] Liu G, Chen Y, Qin X (2014). Transforming rural health care through information technology: an interventional study in China. Health Policy Plan.

[6] Buntin MB, Jain SH, Blumenthal D (2010). Health information technology: laying the infrastructure for national health reform. Health Aff (Millwood).

[7] Li C, Xu X, Zhou G, He K, Qi T, Zhang W, Tian F, Zheng Q, Hu J (2019). Implementation of National Health Informatization in China: Survey About the Status Quo. JMIR Med Inform.

[8] Chen M (2016). Theory and Method of Regional Population Health Informatization.

[9] (2021). National Health Commission, People's Republic of China. Guiding Opinions on Strengthening Health Informatization Construction.

[10] (2013). National Health Commission, People's Republic of China. Guiding Opinions on Accelerating the Information Construction of Population Health.

[11] Hu J, Zhang L, Gu L, Meng Q, Hou Y, Hu J (2014). Health information interoperability and standard system—Practice of China. Health Policy and Technology.

[12] (2017). National Health Commission, People's Republic of China. National Population Health Informatization Development Plan for 13th Five-Year Plan.

[13] Zhao F, Zhang P, Zhang Y, Ma Z (2020). Time to lead the prevention and control of public health emergencies by informatics technologies in an information era. J Biosaf Biosecur.

[14] Wang CJ, Ng CY, Brook RH (2020). Response to COVID-19 in Taiwan: Big Data Analytics, New Technology, and Proactive Testing. JAMA.

[15] Ye Q, Zhou J, Wu H (2020). Using Information Technology to Manage the COVID-19 Pandemic: Development of a Technical Framework Based on Practical Experience in China. JMIR Med Inform.

[16] National Health Commission Statistical Information Center of the People's Republic of China (2019). Survey Report on National Health Informatization.

[17] Mäenpää T, Asikainen P, Gissler M, Siponen K, Maass M, Saranto K, Suominen T (2011). Outcomes assessment of the regional health information exchange: a five-year follow-up study. Methods Inf Med.

[18] Mäenpää Tiina, Suominen T, Asikainen P, Maass M, Rostila I (2009). The outcomes of regional healthcare information systems in health care: a review of the research literature. Int J Med Inform.

[19] (2020). General Office of the National Health Commission, People's Republic of China. Notice of the General Office of the National Health Commission on Informatization Support for Normalized Epidemic Prevention and Control.

[20] Sittig DF, Singh H (2020). COVID-19 and the Need for a National Health Information Technology Infrastructure. JAMA.

[21] Iakovidis I (1998). Towards personal health record: current situation, obstacles and trends in implementation of electronic healthcare record in Europe1Disclaimer: The view developed in this paper is that of the author and does not necessarily reflect the position of the European Commission. International Journal of Medical Informatics.

[22] (2019). Division of Primary Health, National Health Commission, People's Republic of China. Notice on Doing a Good Job in Basic Public Health Service Projects in 2019.

[23] Shen J, Li L (2012). Considerations on rights related to electronic health records. Chinese Journal of Health Informatics and Management.

[24] Meng Q, Ye Q, Hu J (2014). Regional Population Health Informatization Construction and Development.

[25] (2013). National Health Commission, People's Republic of China. Answers to the Guiding Opinions on Accelerating the Construction of Population Health Informatization.

[26] (2020). National Health Commission, People's Republic of China. Opinions on strengthening the construction of the national health information standardization system.

[27] Hu H, Qin P, Lei X (2019). The Development Process and Outlook of National Health Informatization in China. J Med Inform.

[28] Wang K, Mao AY, Meng YL (2019). Development history, current situation, problems and strategies of public health system construction in China. Chin J Public Health.

[29] (2007). Outline of National Health Informatization Development Plan 2003-2010. Health Supervision Center, National Health Commission, People's Republic of China.

[30] Cresswell K, Sheikh A, Franklin BD, Krasuska M, Nguyen HT, Hinder S, Lane W, Mozaffar H, Mason K, Eason S, Potts HWW, Williams R (2020). Theoretical and methodological considerations in evaluating large-scale health information technology change programmes. BMC Health Serv Res.

[31] McCall B (2020). COVID-19 and artificial intelligence: protecting health-care workers and curbing the spread. Lancet Digit Health.

[32] Samra H, Li A, Soh B, Zain MA (2020). Utilisation of hospital information systems for medical research in Saudi Arabia: A mixed-method exploration of the views of healthcare and IT professionals involved in hospital database management systems. Health Inf Manag.

[33] Lee J (2012). EMR management system for patient pulse data. J Med Syst.

[34] Zhou G, Xu X, Hu J (2019). Health Informatization: Development, Characteristics and Prospects. Chinese Journal of Health Informatics and Management.

[35] (2020). National Health Commission, People's Republic of China. Reply to Recommendation No. 8113 of the Second Session of the Thirteenth National People's Congress.

[36] National Health Commission, People's Republic of China (2019). China Health Statistical Yearbook.

[37] Hao XN, Ma CY, Lu ZY (2020). The Effects and Problems on the Reform of Primary Health Informatization in China. Health Economics Research.

[38] (2019). China Hospital Information Management Association. Report on the Situation of Hospital Informatization in China in 2018-2019.

[39] (2019). China Hospital Information Management Association. Research on the fund investment mechanism of regional universal health informatization (III).

[40] Guo Minjiang, Wang Bingqian, Li Juan (2017). International experience of government and social resource cooperation mechanisms in health informatization and its enlightenment. Chinese Health Resources.

[41] Xia L, Qi X, Meng Y (2013). Human resources for public health informatization in CDCs at provincial and municipal levels. Chin J Public Health.

[42] Hong Z, Li N, Li D, Li J, Li B, Xiong W, Lu L, Li W, Zhou D (2020). Telemedicine During the COVID-19 Pandemic: Experiences From Western China. J Med Internet Res.

[43] Hasnain M, Pasha MF, Ghani I (2020). Combined measures to control the COVID-19 pandemic in Wuhan, Hubei, China: A narrative review. J Biosaf Biosecur.

[44] Sjödin Henrik, Johansson AF, Brännström Åke, Farooq Zia, Kriit Hedi Katre, Wilder-Smith Annelies, Åström Christofer, Thunberg Johan, Söderquist Mårten, Rocklöv Joacim (2020). COVID-19 healthcare demand and mortality in Sweden in response to non-pharmaceutical mitigation and suppression scenarios. Int J Epidemiol.

[45] Kho J, Gillespie N, Martin-Khan M (2020). A systematic scoping review of change management practices used for telemedicine service implementations. BMC Health Serv Res.

[46] Song X, Liu X, Wang C (2020). The role of telemedicine during the COVID-19 epidemic in China-experience from Shandong province. Crit Care.

[47] Liu L, Gu J, Shao F, Liang X, Yue L, Cheng Q, Zhang L (2020). Application and Preliminary Outcomes of Remote Diagnosis and Treatment During the COVID-19 Outbreak: Retrospective Cohort Study. JMIR Mhealth Uhealth.

[48] (2020). National Health Commission, People's Republic of China. The regular press conference of the National Health Commission on September 9, 2020 introduced the typical cases of the application and development of national health informatization.

[49] (2019). National Health Commission, People's Republic of China. Standardized maturity assessment of national medical and health information interconnection was carried out smoothly.

[50] (2009). Ministry of Health, People's Republic of China. Notice of the General Office of the Ministry of Health on the Issuance of the Guidelines for the Construction of Regional Health Information Platform Based on Health Records (Trial).

[51] Farokhzadian J, Khajouei R, Hasman A, Ahmadian L (2020). Nurses' experiences and viewpoints about the benefits of adopting information technology in health care: a qualitative study in Iran. BMC Med Inform Decis Mak.

[52] Han Y, Lie RK, Guo R (2020). The Internet Hospital as a Telehealth Model in China: Systematic Search and Content Analysis. J Med Internet Res.

[53] Jiang K, Feng J, Su Y (2020). Prevention and Control Measures of Hospital Informatization from the Perspective of 'Fighting Epidemic'. Health Economics Research.

[54] Ina K, Furuta R, Kayukawa S, Iwasaki M, Sirokawa Y, Koga C, Hibi S, Kabeya M, Yuasa S, Tomomatsu Y, Kataoka T (2018). Medical and dental cooperation using VPN with IPsec in the hospital without dentists. Annals of Oncology.

[55] Ratwani RM, Reider J, Singh H (2019). A Decade of Health Information Technology Usability Challenges and the Path Forward. JAMA.

