# Supplementary material for: Development, Status Quo, and Challenges to China’s Health Informatization During COVID-19: Evaluation and Recommendations
Source: J Med Internet Res. 2021 Jun 17;23(6):e27345. doi: 10.2196/27345 (PMC8213061; doi:10.2196/27345)
Supplement: Multimedia Appendix 1 [file jmir_v23i6e27345_app1.docx]

Multimedia Appendix 1. Data sources on the development and status quo of China’s health informatization from government, commercial, and public welfare sources and websites; academic papers; public reports; institutional reports; and fieldwork.

| Perspective | Contents | Data sources |
| --- | --- | --- |
| Health information  infrastructure | Development of health information platforms and health information databases | Policy documents published by the Ministry of Health, the National Health and Family Planning Commission (NHFPC) and the National Health Commission (NHC) in China |
|  |  | NHFPC’s National Population Health Informatization Development Plan  China’s 13th Five-Year Plan |
|  |  | The Plan of Healthy China 2030 |
|  |  | Researches from the Center for Health Statistics and Information, NHFPC |
|  |  | Academic papers |
|  | The construction rate of provincial, municipal and county-level health information platforms | 2019 National Health Informatization Survey Report published by the NHC  2018 Regional Health Informatization Survey |
|  | The average construction rate of three major databases | 2019 National Health Informatization Survey Report published by the NHC  2018 Regional Health Informatization Survey |
| Information technology applications | The development history of health informatization in China | Outline of National Health Informatization Development Plan 2003-2010 |
|  |  | NHFPC’s National Population Health Informatization Development Plan  China’s 13th Five-Year Plan  Policy documents published by the Ministry of Health, the NHFPC and NHC in China |
|  |  | Researches from the Institute of Medical Information, Chinese Academy of Medical Sciences |
|  |  | Academic papers |
|  | IT application rates on health information platforms and in hospitals | 2019 National Health Informatization Survey Report published by the NHC  2018 Regional Health Informatization Survey  2018 Hospital Informatization Survey |
|  | IT applications in COVID-19 prevention and control | News, reports and information from health authorities, hospitals, and high-tech companies including Baidu, Alibaba and Tencent |
|  |  | Academic papers |
| Financial and intellectual investment | Governmental investment | China’s 12th Five-Year Plan  2017 National Health Informatization Survey conducted by the NHFPC  National Health Security Informatization Project approved by the National Development and Reform Commission |
|  |  | National hospital informatization projects approved by the NHFPC |
|  |  | Policy documents released by the Ministry of Finance, the NHFPC and the NHC in China  Researches from Center for Health Statistics and Information, NHC  NHFPC’s National Population Health Informatization Development Plan  China’s 13th Five-Year Plan  2019 China Health Statistical Yearbook |
|  | Hospital informatization investment | Policy documents published by the NHC  Researches from Center for Health Statistics and Information, NHC  2019 National Health Informatization survey report published by the NHC  2018 Hospital Informatization Survey  2019 CHIMA (China Hospital Information Management Association) survey |
|  | The quantity and quality of health informatization personnel in information departments of administrations and hospitals | 2019 National Health Informatization survey report published by the NHC  2018 Regional Health Informatization Survey  2018 Hospital Informatization Survey |
|  |  | NHFPC’s National Population Health Informatization Development Plan  China’s 13th Five-Year Plan |
|  |  | Academic papers |
| Health resource allocation | The construction strategies of health informatization | Policy documents published by the State Council of the People’s Republic of China  Researches from the Center for Health Statistics and Information, NHFPC |
|  |  | Academic papers |
|  | Medical resource allocation during COVID-19 | News, reports and information from websites of the NHC and hospitals |
|  |  | Academic papers |
| Standard system | The development history of standard system | NHFPC’s National Population Health Informatization Development Plan  China’s 13th Five-Year Plan |
|  |  | Policy documents published by the Ministry of Health, the NHC, and the Special Committee on Health Information Standards  The National Medical Health Information System’s Interconnection and Interoperability Standardization Certification |
|  | The formulation of information standard system and security system | 2019 National Health Informatization survey report published by the NHC  2018 Regional Health Informatization Survey  2018 Hospital Informatization Survey  2018 National Medical Health Information System’s Interconnection and Interoperability Standardization Certification  2017 National Medical Health Information System’s Interconnection and Interoperability Standardization Certification  Policy documents published by the NHC |
|  | The evaluation of information standard system and security system | 2019 National Health Informatization survey report published by the NHC  2018 Regional Health Informatization Survey  2018 Hospital Informatization Survey |
|  | Reforms of national health information standardization system during COVID-19 | The 2020 Notice on Strengthening the National Health Information Standardization System  National Public Health Informatization Construction Standard and Specification  National Hospital Informatization Construction Standard and Specification  Policy documents published by the NHC |
| internet plus health care service pattern | The applications and achievements of internet plus health care in COVID-19 | Information from the NHC’s press conference |
|  |  | News, reports and information from health authorities, hospitals, and high-tech companies including Baidu, Alibaba and Tencent |
|  |  | Academic papers |
